# Costs of life - Dynamics of the protein inventory of *Staphylococcus aureus* during anaerobiosis

**DOI:** 10.1038/srep28172

**Published:** 2016-06-27

**Authors:** Daniela Zühlke, Kirsten Dörries, Jörg Bernhardt, Sandra Maaß, Jan Muntel, Volkmar Liebscher, Jan Pané-Farré, Katharina Riedel, Michael Lalk, Uwe Völker, Susanne Engelmann, Dörte Becher, Stephan Fuchs, Michael Hecker

**Affiliations:** 1 Institute of Microbiology, Ernst-Moritz-Arndt-University Greifswald, F.-L.-Jahn-Strasse 15, D-17487 Greifswald, Germany; 2 Institute of Biochemistry, Ernst-Moritz-Arndt-University Greifswald, Felix-Hausdorff-Strasse 4, D-17487 Greifswald, Germany; 3 Department of Mathematics and Informatics, Ernst-Moritz-Arndt-University Greifswald, Walther-Rathenau-Strasse 47, D-17487 Greifswald, Germany; 4 Interfaculty Institute for Genetics and Functional Genomics, Ernst-Moritz-Arndt-University Greifswald, F.-L.-Jahn-Strasse 15 a, D-17487 Greifswald, Germany; 5 Institute of Microbiology, Technical University Braunschweig, Inhoffenstrasse 7, D-38124 Braunschweig, Germany; 6 Helmholtz Institute for Infection Research, Microbial Proteomics, Inhoffenstrasse 7, D-38124 Braunschweig, Germany; 7 Robert Koch Institute, FG13 Nosocomial Pathogens and Antibiotic Resistance, Burgstrasse 37, D-38855 Wernigerode, Germany

## Abstract

Absolute protein quantification was applied to follow the dynamics of the cytoplasmic proteome of *Staphylococcus aureus* in response to long-term oxygen starvation. For 1,168 proteins, the majority of all expressed proteins, molecule numbers per cell have been determined to monitor the cellular investments in single branches of bacterial life for the first time. In the presence of glucose the anaerobic protein pattern is characterized by increased amounts of glycolytic and fermentative enzymes such as Eno, GapA1, Ldh1, and PflB. Interestingly, the ferritin-like protein FtnA belongs to the most abundant proteins during anaerobic growth. Depletion of glucose finally leads to an accumulation of different enzymes such as ArcB1, ArcB2, and ArcC2 involved in arginine deiminase pathway. Concentrations of 29 exo- and 78 endometabolites were comparatively assessed and have been integrated to the metabolic networks. Here we provide an almost complete picture on the response to oxygen starvation, from signal transduction pathways to gene expression pattern, from metabolic reorganization after oxygen depletion to beginning cell death and lysis after glucose exhaustion. This experimental approach can be considered as a proof of principle how to combine cell physiology with quantitative proteomics for a new dimension in understanding simple life processes as an entity.

The publication of the first entire genome sequence of the bacterium *Haemophilus influenzae* in 1995 marked a turning point in life sciences because it provided the first blueprint of a self-replicating (micro)organism^1^. Since then the genome sequences of thousands of organisms including humans have been published. It turned out, however, that genome sequencing is only the first step on the way to a comprehensive understanding of cellular functions on molecular level. In the second step functional genomics is required to translate this blueprint of virtual life into real cellular physiology. Within the ensemble of OMICs techniques proteomics holds a privileged position because proteins execute and regulate most cellular functions in all organisms. Metabolite data, in turn, provide a readout of the function and activity of proteins. Because of their low complexity, bacteria are particularly suited to study this translation of the genome sequence into real life processes since the protein inventory of a bacterial cell consists of only a few hundreds up to a few thousands of proteins which can be efficiently handled by current proteomics techniques.

To provide the protein inventory of an organism is not only one of the important achievements of life sciences in the post genomic era but also the next crucial step after genome sequencing on the long way from the genome sequence to comprehension of cell function. Meanwhile the majority of proteins synthesized in bacteria are identified for model microorganisms such as *Bacillus subtilis*^2^,^3^, *Escherichia coli*^4^,^5^, *Leptospira interrogans*^6^,^7^, *Mycoplasma pneumoniae*^8^,^9^, *Mycobacterium tuberculosis*^10^,^11^, *Saccharomyces cerevisiae*^12^,^13^, and *Staphylococcus aureus*^14^. Becher and co-workers identified the greater part of proteins synthesized in exponentially growing and glucose-starved cells of *S. aureus*^14^. *S. aureus* was selected as a model in this study, too, because of its medical importance, which is illustrated by the broad range of infections that are caused by *S. aureus* and that are increasingly refractory to treatment because of the multiple resistant clones.

Here, we provide absolute quantitative protein data to monitor the dynamics of the protein inventory of *S. aureus* to an infection-related stimulus. This can be considered as a new and promising approach to understand *S. aureus*’ pathophysiology and infection biology. Absolute protein quantification is crucial to obtain a real mass balance of what is going on in the cell in response to external stimuli such as stress and starvation. In the past years mass spectrometry based methods have been developed for absolute protein quantification^15^,^16^,^17^,^18^. Combinations of different approaches were established for the determination of protein amounts on a global scale for model bacteria^6^,^7^,^10^,^19^. Protein identification rates and sequence coverage were improved with the introduction of data-independent acquisition methods such as LC-MS^E ^20,21. Complemented with the Hi3 absolute quantification^18^ even more comprehensive quantitative data sets can be generated^22^. However, there are only very few studies that combine the dynamics of the entire protein inventory with interpretation of basic physiological processes aiming at the understanding of cell physiology as an entity, the main goal of this study^2^,^3^,^10^. In contrast, numerous studies focus on *S. aureus* regarding pathogenicity and virulence factors such as adhesins and invasins, toxins, superantigens, immune evasion proteins, and antibiotic resistance mechanisms^23^. But only limited information is available on basic cellular physiology compared to well-known model organisms such as *Bacillus subtilis* or *Escherichia coli*. The survival and persistence in the human host is mainly dependent on the physiological fitness including the adaptation to stress and starvation stimuli.

In 2007 we studied the immediate reactions of *S. aureus* COL to oxygen starvation on transcriptomic and protein synthesis level^24^. In addition to DNA array techniques radioactive pulse-labeling of proteins was used to visualize early changes in the gene expression patterns immediately after the transition to oxygen starvation. However, the number of protein molecules that execute cell functions as well as the adaptation to prolonged anaerobic periods was not yet considered^24^,^25^.

In this study we analyzed the dynamics of the protein inventory during long-term adaptation to oxygen starvation with the focus on quantitative proteomics. When invading different host tissues, *S. aureus* encounters low oxygen levels as one of the most important growth-limiting factors^26^,^27^. Thus, the observed adaptation strategies are crucial for *S. aureus* because oxygen limitation is one of the dominating stimuli in many infection-related settings. Using a combination of ion mobility LC-MS^E^ (LC-IMS^E^) and Hi3 quantification, protein copy numbers of 1,168 cytosolic proteins were determined and complemented with concentrations of extra- and intracellular metabolites to provide a complete picture on the physiology of this long-term adaptation process. This is one of the first studies linking absolute protein amounts to crucial adaptational and physiological processes of a pathogenic microorganism. Thus, at least 1,200,000 cytoplasmic protein molecules represent the machinery of life in a single *S. aureus* cell with a volume of 320 attolitre in average. Quantitative Voronoi treemaps were used for visualization of the cellular investment in proteins involved in fundamental processes of life under oxygen limitation. Our experimental approach can be regarded as a proof of principle study that illustrates how quantitative proteomics and metabolome data can be integrated to come to a new dimension in understanding simple life processes as an entity. Such studies may thus pave the way towards a higher quality in understanding pathophysiology, opening new avenues for infection-related research on this and other crucial pathogens.

## Results and Discussion

### Three different physiological states can be defined by the availability of oxygen and glucose

**Figure 1:**
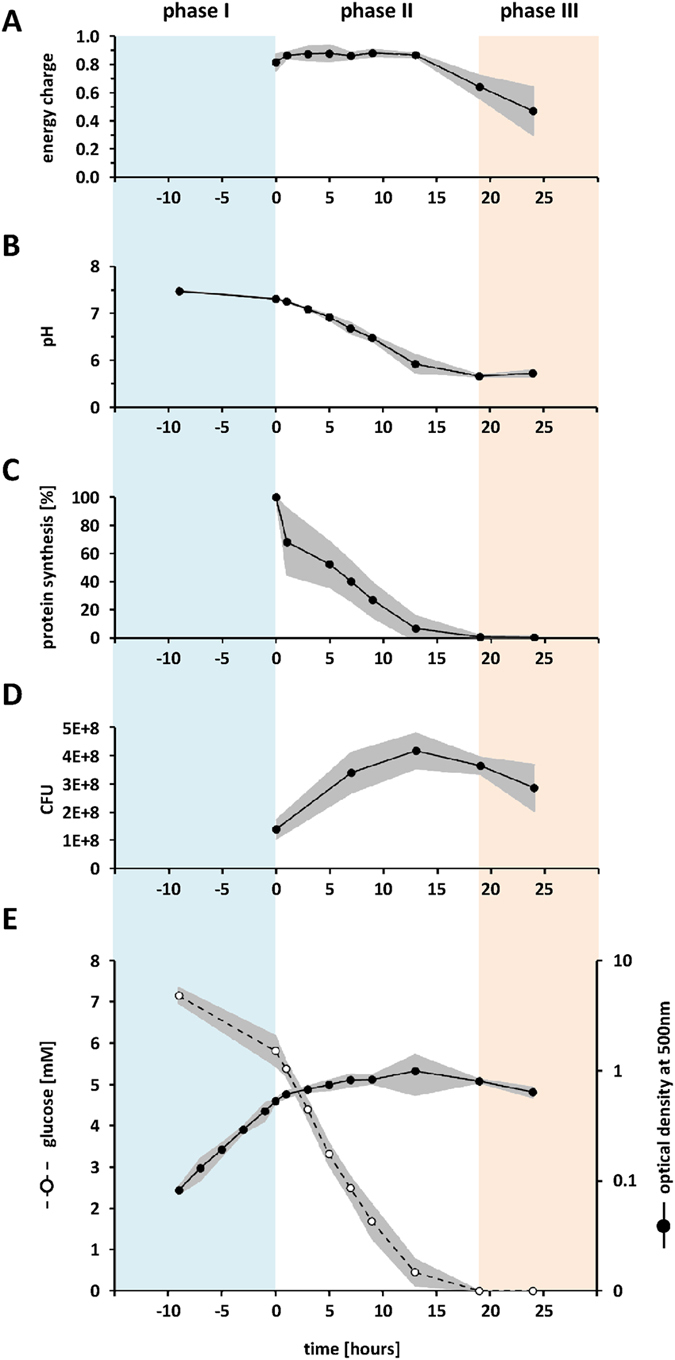
Growth parameters of *S. aureus* COL during long-term anaerobic conditions. Cells were grown in chemically defined medium until mid-exponential phase and then transferred to anaerobic conditions. The different physiological conditions are indicated on the top: Phase I (blue) represents aerobic growth in the presence of glucose, phase II (white) means anaerobic growth in the presence of glucose, phase III (orange) shows growth in the absence of glucose and oxygen. Energy charge (**A**) was calculated based on intracellular concentrations of AMP, ADP, and ATP. The pH of the medium (**B**) has been monitored during growth. Newly synthesized proteins were labelled by L-[^35^S]-methionine (**C**) and radioactive incorporation determined using whole cell extracts (see Supplementary Methods). Bacterial survival is shown in colony forming units (**D**). Extracellular glucose concentration [mM] has been measured using ^1^H-NMR spectroscopy (**E**). Bacterial growth is shown as optical densities at 500 nm in a semi-logarithmic scale. Confidence intervals were calculated from three replicates for each data point and are shown as grey area.

*S. aureus* was first cultivated in the presence of oxygen and high levels of glucose (7.2 mM). At mid-exponential growth phase (OD_500_ _nm_ 0.5) cells were shifted to anaerobic conditions and cultivated for further 24 hours. During aerobic cultivation *S. aureus* was exponentially growing with a growth rate [μ] of 0.207 h^−1^ (Fig. 1E). As expected, growth rate significantly dropped after oxygen depletion (μ = 0.049 h^−1^). Interestingly, cells were not able to use alternative energy sources such as amino acids after exhaustion of glucose and, thus, started dying as determined by a clear decrease of CFU (Fig. 1D).

Thus, at least three main growth phases have been considered by our experimental setup: (i.) exponential growth in the presence of oxygen and glucose (0–t_0_), (ii.) slow growth in the absence of oxygen and presence of glucose (t_1_–t_13_), and (iii.) cessation of growth, cell death and lysis in the absence of oxygen and glucose (t_13_–t_24_).

### The dynamics of the *S. aureus* proteome and metabolome in response to oxygen and glucose starvation

In total, 1,390 proteins have been identified in at least two biological replicates at one or more time points of the growth curve (Supplementary Data 1). This includes 222 non-cytoplasmic proteins (mainly membrane-bound and -associated proteins) which have been excluded from further analyses since these mainly hydrophobic proteins cannot be completely recovered by the applied approach^14^. Absolute protein concentrations have been used to calculate molecule numbers per cell for 1,168 cytosolic proteins which corresponds to 64% of the cytosolic *S. aureus* proteome predicted by LocateP^28^ (Supplementary Data 1). The real coverage is even higher considering that not all *S. aureus* genes are expressed during the conditions analyzed. The dynamic range covering proteins present in thousands or only few copies per cell is remarkably high (for example: 20,000 molecules per cell of GapA1 and 20 molecules per cell of ArcB1 under anaerobic conditions). Additionally, concentrations of intra- and extracellular metabolites were determined using ^1^H-NMR spectroscopy, GC/MS and LC/MS. In total, time-resolved concentration profiles of 29 extracellular and 78 intracellular metabolites have been integrated (Supplementary Data 2).

### Vegetative proteins dominate the cytoplasmic proteome during phase I (aerobic growth in the presence of glucose)

**Figure 2:**
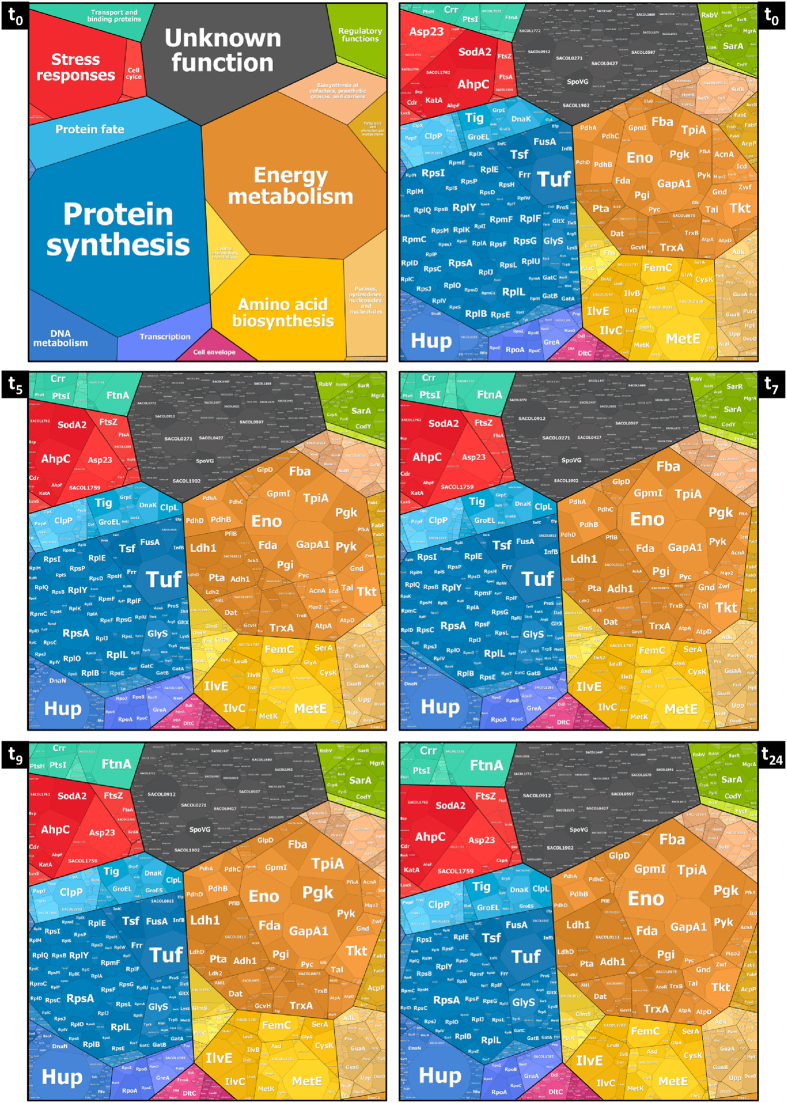
Quantity-dependent visualization of dynamics in the *S. aureus* COL protein inventory during long-term anaerobiosis. Normalized protein amounts are displayed in an area-encoded fashion using Voronoi treemaps. The upper leftmost treemap shows functional protein classification based on TIGR Roles and manual curation. The different sample points can be grouped into phase I (aerobic growth in the presence of glucose; t_0_), phase II (anaerobic growth in the presence of glucose; t_5_, t_7_, t_9_), and phase IIII (anaerobic growth in the absence of glucose).

In total, 996 cytosolic proteins were quantified when both, glucose and oxygen, are available (Supplementary Data 1). Based on these data about 1,200,000 protein molecules are packed into only 0.32 μm^3^ of an exponentially growing *S. aureus* cell (Supplementary Table S1). With 190,000 copies per cell (16%) the 53 identified ribosomal proteins account for the largest proportion followed by proteins involved in amino acid metabolism (114,000 copies per cell; 10%), glycolysis (83,000 copies per cell; 7%), protein folding, repair and degradation (52,000 copies per cell; 4%), and TCA cycle (15,000 copies per cell; 1%) (Fig. 2).

Surprisingly, only three (alanine, serine, and aspartate) of the 15 amino acids provided were consumed by *S. aureus* during exponential growth in significant quantities (Supplementary Fig. S1). This closely correlates with the high amount of enzymes involved in the conversion of these amino acids to lysine, threonine, cysteine, methionine, glycine, valine, leucine, and isoleucine (98,500 molecules per cell; Fig. 4).

**Figure 3:**
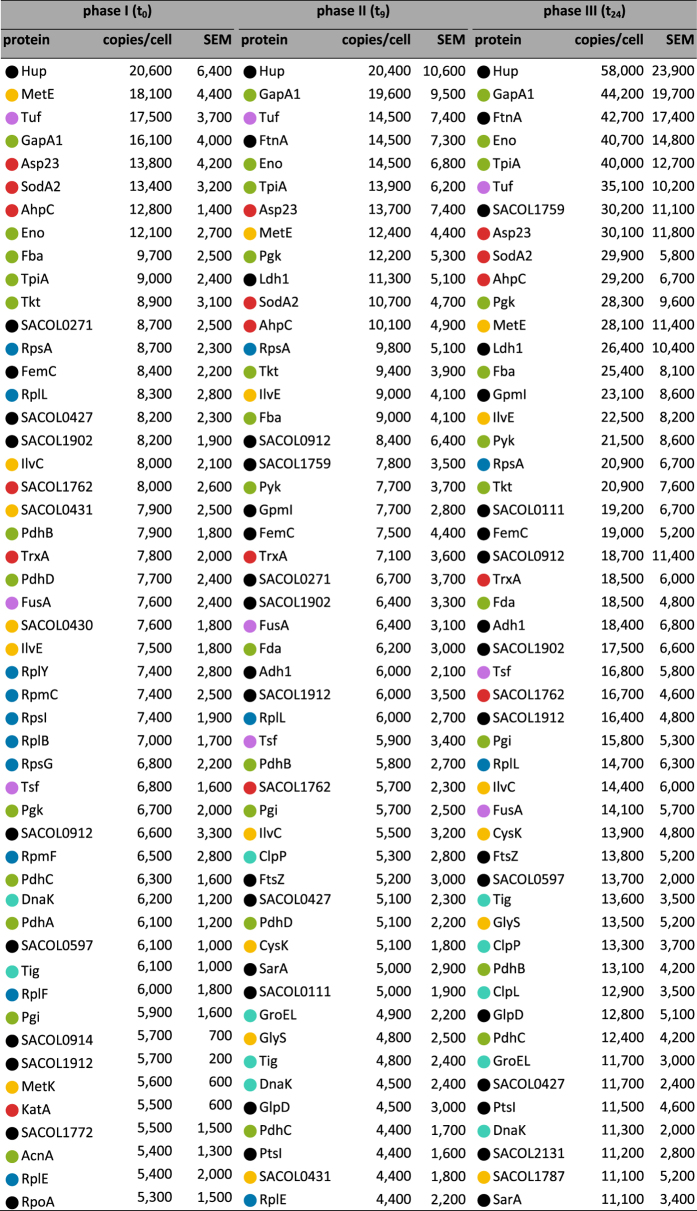
Molecule numbers per cell and standard errors of the mean (SEM) of the 50 most abundant proteins that can be grouped into different physiological classes (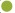 glycolysis, pentose phosphate pathway, and TCA cycle; 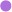 translation; 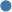 ribosomes; 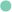 protein folding and degradation; 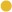 amino acid metabolism, 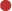 stress responses; 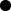 misc). All values are rounded to full hundred.

**Figure 4:**
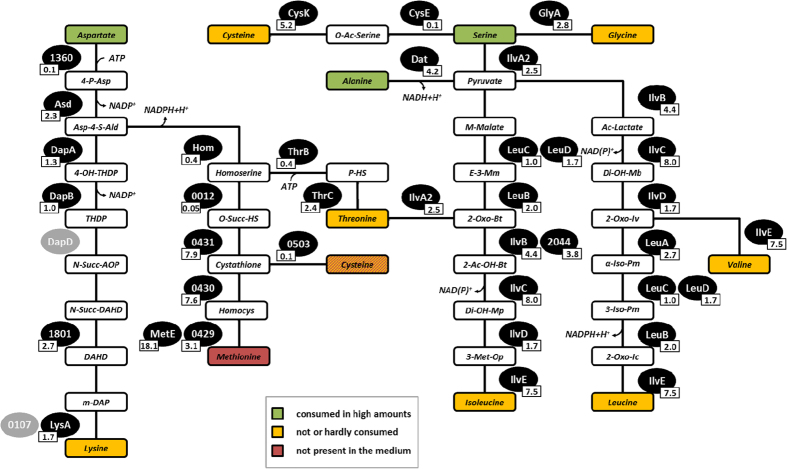
Conversion of selected amino acids in *S. aureus* COL under aerobic conditions (t_0_). Shown are the involved enzymes with their respective copy number per cell in thousands during mid-exponential growth. Proteins with grey background were not identified. In aqueous solution cysteine reacts to a large part to cystine. For both metabolites no extracellular concentrations were evaluable due to strong pH dependent shifts in the NMR spectra. 4-P-Asp = 4-Phospho-L-aspartate; Asp-4-S-Ald = Aspartate-4-Semialdehyde; 4-OH-THDP = 4-Hydroxy-2,3,4,5-tetrahydrodipicolinate; THDP = 2,3,4,5-tetrahydrodipicolinate; N-Succ-AOP = N-Succinyl-2-amino-6-oxopimelate; N-Succ-DAHD = N-Succinyl-2,6-diaminoheptanedioate; DAHD = 2,6-Diaminoheptanedioate; meso-2,6-Diaminopimelate; O-Succ-HS = O-Succinylhomoserine; Homocys = Homocysteine; P-HS = O-Phospho-homoserine.

In the presence of oxygen and high levels of glucose, glycolytic enzymes belong to the highest abundant proteins (Fig. 3) which allow rapid degradation of glucose (0.34 μM × h^−1^ × Δcell number^−1^) indicating that a significant part of the ATP is provided by substrate phosphorylation^29^. Interestingly, enzymes of the preparatory part of glycolysis (Glk, Pgi, PfkA, Fba) are present in lower amounts compared to enzymes employed in the pyruvate converting branch (TpiA, GapA1, Pgk, Pgm, Eno, Pyk) (Supplementary Fig. S2). This correlates well with the number of molecules channeled through the respective branches (preparatory phase: 1x C_6_; pyruvate converting reactions: 2x C_3_).

**Figure 5:**
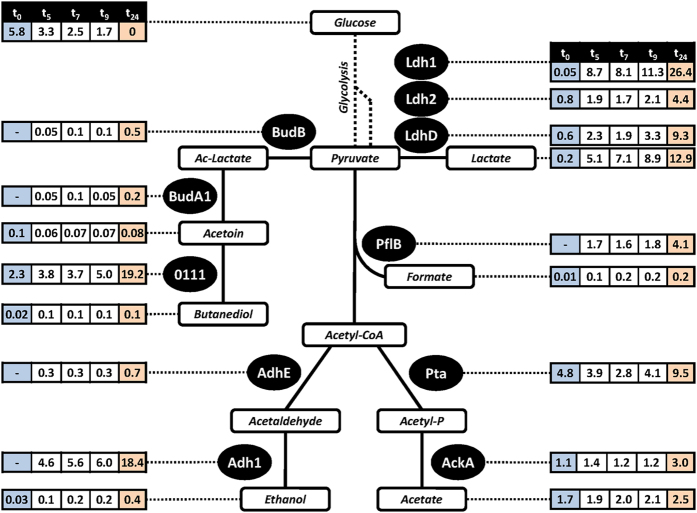
Protein amounts and exometabolite concentrations related to fermentation and overflow metabolism in *S. aureus* COL upon anaerobic conditions. The absolute protein abundances (protein copies per cell; numbers are given in thousands) and the extracellular metabolite concentrations (in mmol/L) are shown for different time points (hours) after oxygen depletion. Colors indicate different physiological conditions: phase I (blue) aerobic in the presence of glucose; phase II (white) anaerobic in the presence of glucose; phase III (orange) anaerobic in the absence of glucose. Ac-Lactate = Acetolactate; Acetyl-P = Acetylphosphate.

The high level of glucose used in this study prevents their complete oxidation and triggers down-regulation of the TCA cycle enzymes (*Crabtree effect*) (Supplementary Fig. S3). Pyruvate is converted to overflow metabolites (mainly acetate) which is secreted into the medium (Fig. 5)^24^,^30^. Involved enzymes are present in high numbers (phosphate acetyltransferase Pta: 4,800 copies per cell; acetate kinase AckA: 1,100 copies per cell). At the end of phase I 1.65 mM acetate has been secreted by *S. aureus* which corresponds to 62% of glucose degraded in this time span (1.34 mM) (Fig. 5, Supplementary Data 2).

In addition to the central metabolic pathways *S. aureus* invests mainly in proteins for growth and reproduction such as DNA metabolism, transcription, and translation during exponential growth (Fig. 2). For instance, with 17,500 copies the translational factor Tuf is one of the most abundant proteins (Fig. 3).

### The protein inventory is completely reorganized in response to oxygen starvation as long as glucose is available (phase II)

Depletion of oxygen induced a major reorganization of the *S. aureus* proteome (Fig. 2). To follow these changes more easily, an animated and zoomable movie has been created based on Voronoi Treemaps ( http://www.protecs.uni-greifswald.de/bionic-vis/timeline/costsoflife.html). As expected, production of glycolytic enzymes was increased even further despite the high level already present in phase I (for example: enolase increased from 12,100 to 14,500 or GapA1 from 16,100 to 19,600 molecules per cell; Fig. S2). This was accompanied by a strong increase in level of fermentation enzymes. With up to 11,300 copies per cell the lactate dehydrogenase Ldh1 is by far the most abundant fermentation enzyme even during long-lasting anaerobiosis. Ldh2 and the D-lactate producing LdhD (SACOL2535) are also produced in higher amounts after oxygen starvation but only to a level of 2,100 and 3,300 molecules per cell, respectively (Fig. 5). In accordance lactate was measured as the main fermentation product of *S. aureus* and is strongly accumulating within the cells (up to 350 nmol/mg CDW) as well as secreted in high amounts (Fig. 5). Thus, lactate is mainly responsible for the significant decrease of extracellular pH from 6.8 to 5.2 (Fig. 1B). Additional fermentation products (ethanol, acetate, formate, 2,3-butanediol) were secreted only in very little amounts which is in accordance with the low level of respective fermentation enzymes (Fig. 5). Pyruvate formate lyase PflB was newly detectable, but only up to 1,800 copies per cell. Other enzymes involved in mixed acid or alternative fermentation pathways were also present in low amounts (AdhE: 300 copies per cell; BudA1 and BudB: less than 100 copies per cell each). The only exceptions were the alcohol dehydrogenase Adh1 (6,000 copies per cell) and the acetoin reductase SACOL0111 (5,000 copies per cell). Our metabolic data indicate that SACOL0111 is responsible for re-utilization of acetoin secreted in phase I. By this acetoin can be converted to 2,3-butanediol which regenerates an additional NAD^+^. Accordingly, a decrease of extracellular concentrations of acetoin and an increase of 2,3-butanediol have been observed within the first three hours of anaerobiosis (Supplementary Data 2).

However, it is still a matter of debate why *S. aureus* prefers lactate fermentation even if the mixed acid fermentation would provide an additional ATP per molecule glucose. Lactate dehydrogenases compete with other fermentation enzymes for pyruvate and NADH+H^+^. As shown here, Ldh1 is by far the most abundant fermentation enzyme in anaerobic *S. aureus* cells. This might be the major factor determining the pyruvate flux under anaerobic conditions as shown for *Lactococcus lactis*^31^. However, activity of PflB is needed because it is essential to supply formate for metabolic processes such as the purine and protein biosynthesis under anaerobic conditions^32^.

Our data suggest that Ldh1 plays anaerobically the central role in maintaining the redox balance in *S. aureus* at least under glucose excess. Mixed acid fermentation may become important when glucose becomes limited or during growth on alternative carbon sources.

In the absence of oxygen (and alternative electron acceptors) and the presence of glucose ATP generation in *S. aureus* is restricted to the level of substrate phosphorylation during glycolysis (*Pasteur effect*) explaining the further increase in glycolytic activity under fermentation conditions (consumption of up to 0.56 μM glucose × h^−1^ × Δcell number^−1^; 165% compared to phase I) compared to the already high level of *S. aureus* growing aerobically with glucose excess. Increased flux through glycolysis ensures maintenance of the cellular energy charge within the physiological range of 0.80 to 0.95^33^ even after several hours of anaerobiosis as long as glucose is present (Fig. 1A). Most interestingly, with the exception of ATP intracellular concentration of nucleotide triphosphate pools (UTP, GTP, and CTP) dropped under anaerobic conditions (Table 1).

Oxygen depletion has also a significant impact on the consumption of available amino acids (Supplementary Fig. S1). Alanine was no longer consumed under anaerobic conditions, but was even secreted as fermentation product (in very small amounts). This might be an indication of pyruvate conversion to alanine which recycles one molecule NADH+H^+^ to NAD^+^. The involved alanine dehydrogenase Ald1 was only accumulating after oxygen depletion (1,400 copies per cell; Supplementary Data 1). Additionally, serine and threonine were degraded anaerobically in high amounts, while isoleucine and lysine were taken up in lower amounts (Supplementary Fig. S1). Notably, TdcB required for the degradation of threonine and serine was exclusively synthesized in oxygen-starved cells (100 copies per cell).

As previously reported, expression level of genes involved in growth and replication significantly decreases after oxygen limitation^24^ which is in accordance with our data. The number of ribosomal proteins and proteins involved in transcription (RNA polymerase) and translation (aminoacyl-tRNA synthetases, translation factors) clearly decreased during long-term anaerobiosis to about 66% of the level measured in growing cells (Fig. 2). This is probably caused by reduced synthesis and dilution of ribosomal proteins and not by proteolysis, since pulse-chase experiments using incorporation of L-[^35^S]-methionine showed that the proteins synthesized in phase I are stable for several hours after shift to anaerobic conditions (Supplementary Fig. S4). Degradation of proteins could be observed only in the late phase II.

### In the absence of glucose and oxygen life processes can no longer be maintained (phase III)

After 13 hours of anaerobic growth glucose was completely taken up by *S. aureus* inducing phase III that is characterized by low metabolic activity, cell death and lysis (Fig. 1). Without oxygen the lack of glucose could not be compensated and the energy charge dropped to 0.4 to 0.6. Carbon sources available after consumption of glucose such as amino acids were not consumed or only at a slight rate which did not prevent the drop in the energy charge (Fig. 1A).

In phase III a further secretion of fermentation products (lactate, formate, ethanol) can be no longer observed indicating the stop of fermentative processes. Accordingly, intracellular levels of NADH+H^+^ increased significantly (Supplementary Fig. S5). Gluconeogenetic enzymes were produced only in low amounts (for example: 300 PckA copies per cell; Supplementary Data 1) indicating that the energetic situation did not allow sufficient investment in gluconeogenesis that would be urgently required for sustaining life processes under these conditions. This is reflected by decreasing levels of glucose-6-phosphate and fructose-6-phosphate, but a relatively constant level of pyruvate (Supplementary Fig. S5).

Furthermore, the absence of glucose led to a derepression of carbon catabolite controlled genes, *e.g.* enzymes involved in arginine deiminase pathway (ArcB1, ArcB2, and ArcC2)^34^. The level of these enzymes, however, was extremely low (only 20 to 200 copies per cell; Supplementary Fig. S6; Supplementary Data 1), probably because of the low energy charge. In the absence of oxygen and glucose *S. aureus* can use arginine as an energy source to generate ornithine, ammonia, carbon dioxide and 1 mol ATP per mol arginine by the deiminase. Indeed, a slight increase of intracellular and extracellular ornithine has been observed when glucose is exhausted (Supplementary Figs S1 and S6; Supplementary Data 2). A strong induction of the deiminase pathway as observed in rich media^34^ failed in the nutrient-reduced synthetic medium used in this study. Thus, ATP produced by the arginine deiminase pathway, probably one of the main ATP sources in the absence of glucose, is not sufficient to keep the energy charge in *S. aureus* high enough to prevent cell death and lysis under the tested conditions. As a consequence of the low energy charge overall protein synthesis measured by L-[^35^S]-methionine incorporation nearly stopped (Fig. 1C).

Thus, energy limitation might be the major factor for loss of viability and the missing fermentative and gluconeogenetic activity just a consequence under the tested conditions.

It has been previously described that in glucose starved cells vegetative proteins no longer active in the non-growing state are strongly degraded if oxygen is available^35^. The amino acids released by proteolysis can serve as energy at sufficient oxygen supply. In the absence of oxygen, amino acids of the medium were not used as energy sources and a degradation of vegetative proteins, an ATP-consuming process, was not found (Supplementary Fig. S4).

### Global starvation responses under anaerobic conditions (in phase II and III)

Next we addressed the question which global stress and starvation responses became active in phase II (reduced growth rate) and phase III (no growth and cell lysis, respectively). Expression of most genes coding for fermentation enzymes is controlled by the redox-sensitive Rex repressor^25^ and can be induced within few minutes after oxygen limitation^24^. Whereas Ldh1 and LdhD are exclusively regulated by Rex, the enzymes involved in mixed acid fermentation (PflB, Adh1, AdhE) are under additional control^25^. For instance, the response regulator SrrA–phosphorylated by its membrane-bound sensor kinase SrrB only in the absence of oxygen–activates the expression of its own operon (*srrAB*) as well as of genes encoding enzymes of the mixed acid fermentation^36^. This is in accordance with the observed relatively low basal level of SrrA under aerobic conditions (300 molecules per cell) and significant increase under anaerobic conditions (up to 1,300 molecules per cell) (Supplementary Table S2).

DNA array studies indicate a weak stringent response in phase II^24^. However, the alarmone ppGpp was absent or at least below the detection limit in this study.

With 118 molecules per cell the alternative sigma factor SigB was already present in significant amounts during exponential growth (t_0_) in our experimental setting. For comparison, the housekeeping sigma factor RpoD was present in 368 copies per cell at t_0_. However, SigB levels did not increase in phase II and III. Interestingly, the anti-anti-sigma factor RsbV was induced in phase III (from 4,400 at t_0_ to 7,100 molecules per cell at t_24_) which might have an effect on SigB activity.

Finally, phase III triggered an activation of the glucose starvation specific CcpA response that is no longer active to repress glucose regulated genes in the absence of glucose. However, the energetic situation only allows a slight activation of CcpA dependent processes shown by the weak activation of the arginine deiminase pathway already discussed^37^,^38^.

### Journey into the unknown: expression profiling of so far functionally uncharacterized proteins

In this proteomics study proteins can be divided into four groups: (i) proteins newly produced in response to oxygen starvation (Supplementary Data 1), (ii) proteins already present in cells grown in the presence of oxygen but provided in higher amounts after oxygen depletion, (iii) proteins whose level remains more or less the same both in the presence and absence of oxygen, and finally (iv) proteins whose level drops in response to oxygen starvation. Many functionally so far uncharacterized proteins can be found in group 1 and 2 (Supplementary Table S3) indicating a function in the adaptation to oxygen starvation. One of the ten most abundant proteins under anaerobic conditions is SACOL1952 (FtnA) whose amount steadily increased from 5,000 copies per cell in phase I to 14,500 and 42,700 copies in phase II and III, respectively (Figs 2 and 3). This protein belongs to the ferritin protein family, which is composed of 24 identical subunits and serves as iron storage and provides intracellular iron reserves^39^. Expression of *SACOL1952* is regulated by PerR^40^ and by the cellular iron level^41^. The calculated number of SACOL1952 molecules and the subunit composition suggests the formation of about 1,800 ferritin complexes after 24 h of anaerobiosis. We suggest that the Fe solubility in the medium increased under anaerobic conditions at acid pH (shift from Fe(III) to Fe(II)) leading to a strong increase in Fe uptake followed by induction of *SACOL1952* expression^41^. However, the expression of other PerR-regulated genes were downregulated which includes genes encoding for the assembly of Fe-S-clusters (*sufC*, *sufD*, *sufS*, *SACOL0917*, *sufB*) no longer synthesized after shift to anaerobic conditions. The stoichiometry of the SufBCD complex is determined as 1:2:1 for *E. coli*^42^. A similar stoichiometry of the Fe-S-cluster assembly proteins was calculated for *S. aureus* COL. The stoichiometries of other protein complexes of *S. aureus* could be determined as well by our approach for absolute protein quantification and are in good agreement with published data from other bacteria (Table 2).

Despite the low energy charge *S. aureus* still synthesized proteins in phase III. One of these proteins is the azoreductase AzoR which might probably be involved in the regeneration of NAD^+^. Although, it is one of the prominent proteins in phase III, the copy number only increased from about 700 copies in phase II to 4,000 in phase III (Fig. 2; Supplementary Data 1).

Another protein with strongly increased levels during oxygen starvation was ClpL (SACOL2563; from 1,100 to 4,000 molecules per cell) but the level of ClpX decreased at the same time (from 1,900 to 900). The function of ClpL in response to oxygen starvation is still a matter of debate and has to be analyzed in follow up-studies.

Notably, the production of the universal stress protein UspA (SACOL1759) was strongly increased in oxygen-starved and even more in oxygen/glucose-starved cells (from 4,800 copies per cell in t_0_ to 7,800 in phase II and 30,200 in phase III) (Fig. 2; Supplementary Data 1). This result fits with the finding that UspA is a general indicator for many stress situations in *S. aureus*^43^.

In addition to these four prominent examples there were 71 proteins with still unknown function that were only present under fermentation conditions, and 33 unknown proteins whose level increased at least two-fold in phase II (Supplementary Table S3). For these proteins a function in the adaptation to oxygen starvation is likely.

### Molecule numbers of transcriptional factors correlate with regulon size

As expected, transcriptional factors belong to the proteins with the lowest molecule numbers per cell (few to several hundreds) (Supplementary Table S2). In total, 57 transcriptional factors and response regulators have been identified and quantified in this study. As one would expect, molecule numbers of regulators of gene expression showed a positive correlation with regulon size of the respective regulator. For instance, CodY and CcpA were among the most abundant transcriptional factors during aerobic growth with 1,900 and 1,300 molecules per cell, respectively. Both control large regulons of approximately 100 and 60 operons, respectively^44^,^45^. Notably, the MarR-type transcriptional regulator MgrA was in our study the most abundant regulator (2,600 molecules per cell; Supplementary Table S2). MgrA is a global regulator that controls expression of about 350 target genes, among them genes that encode for virulence factors, autolysis and capsule production. Moreover, it regulates the expression of efflux transporters that lead to an increased resistance to antibiotics^46^. A strong level increase in response to oxygen starvation was found for CodY, YycF, SACOL1541, PyrR, SrrA, and SaeR (Supplementary Table S2). Regulators controlling only small regulons were found only in low amounts. For instance, CtsR controlling only five operons is present only in 50 to 100 copies per cell. The regulator GraR (SACOL0716) that regulates the expression of about 30 genes was present with 50 copies per cell in phase I.

### Costs of life - Comparative assessment of cellular investments in the protein inventory during the different physiological states

Combining the changes of molecules per cell between two time points with the specific molecular weight the cumulative cellular investment in the respective protein species can be estimated. As expected, exponentially growing cells invested mainly in proteins involved in energy metabolism (24%) and protein synthesis (22%), but also in gene expression including transcription, translation, protein fate and quality control, and many others (Supplementary Fig. S7). This pattern changed clearly after oxygen depletion when protein synthesis rate dropped down to 50% after the first five hours of anaerobiosis compared to the rate observed in aerobically growing *S. aureus* cells (Fig. 1C). Investment in proteins involved in glycolysis (Pyk, Pgk, TpiA, Pgm) and fermentation (Ldh1, PflB, Adh1, Ald1) had highest priority indicating that generation of ATP and NAD^+^ are the growth limiting processes during the early stage of anaerobic adaptation (Supplementary Fig. S7; Fig. 3). After glucose exhaustion cells are not able to compensate the lack of energy. Investment in ribosomal proteins reaches a new minimum of 4.7% (8.3% during exponential growth at t_0_). Most resources were used by *S. aureus* in this phase to maintain the anaerobic energy metabolism (18%). However, investment calculations in this phase are prone to more uncertainty because of partial cell death and lysis.

Our results show that the presentation of the entire proteome and its dynamics in relation to environmental changes is the next crucial step after genome sequencing towards the understanding of life processes as an entity. This dataset generated *in vitro* with the oxygen starvation indicator proteins may be helpful for the interpretation of the life style of *S. aureus* grown under *in vivo* conditions (see^47^). Furthermore, our integrated and quantitative Multi-OMICS approach may serve as a starting point for a systems biology application and consideration in near future. With the identification of the protein inventory of a cell, however, we are just at the beginning of a long way towards the elucidation of the molecular mechanisms of simple life. The next step that follows is to understand how the hundreds of different protein molecules released from the ribosome organize life of a bacterial cell. Right after their emergence from the ribosome tunnel, they find their partners and their final destination, organize huge protein networks, they are modified, damaged by external stimuli, protected or repaired and in hopeless cases or in cases of “unemployment” degraded. To understand this dynamics of the protein fate from birth at the ribosome to death at the proteomics scale belongs to the greatest challenges for future research from bacteria to men. Quite clearly, *S. aureus* appears to be an excellent model system to tackle this crucial question of life sciences. This new quality of knowledge will lead to a new dimension in the understanding of cell physiology and pathophysiology including host-pathogen interaction which is required to better understand and finally better combat this malicious human pathogen.

## Material and Methods

### Growth conditions and sampling points

*S. aureus* COL^48^ was grown under aerobic conditions in a modified chemically defined medium (CDM^30^) at 37 °C under vigorous agitation. At mid-log phase (OD_500_ _nm_ 0.5) cells were shifted to anaerobic conditions by transferring the culture to 50 mL Falcon tubes which were completely filled with bacterial culture, tightly closed, and incubated under vigorous agitation at 37 °C. Samples were taken immediately before shift (control t0) and 1, 3, 5, 7, 9, 13, 19, and 24 h (t1, t3, t5, t7, t9, t13, t19, t24, respectively) after shift to anaerobic conditions.

### Preparation of cytoplasmic protein extracts

Before and at different time points after shift to anaerobic conditions 50 mL of the bacterial culture were harvested by centrifugation (4 °C, 8,500 rpm, 10 min), the cell pellets were washed twice with 1 mL TE buffer (10 mM Tris, 1 mM EDTA, pH 8.0), resuspended in 1 mL TE buffer and transferred in a 2 mL screw cap micro tube containing 500 μL glass beads with a diameter of 0.1 mm. Mechanical disruption of the cells was achieved using a Precellys 24 homogenizator (PeqLab, Germany; 3 × 30 sec at 6,800 rpm). Cell debris and glass beads were separated from the proteins by centrifugation for 10 min at 4 °C at 15,000 rpm followed by a second centrifugation step to remove insoluble and aggregated proteins (30 min, 4 °C, 15,000 rpm). Determination of protein concentration by the ninhydrin-based assay^49^ was performed as described earlier^19^.

### Sample preparation for global label-free absolute quantification

In-solution digestion of protein extracts with trypsin was performed as described previously^50^. Desalting of peptides prior to mass spectrometry analysis using stage tips was achieved using a standard protocol^51^. For absolute quantification, a tryptic digest of yeast alcohol dehydrogenase (ADH1, Waters, USA) was added into the samples to final concentration of 50 fmol/μL.

### LC/MS setup for label-free quantification (LC-IMS^E^)

The nanoACQUITY™ UPLC™ system (Waters, USA) was used to separate the peptide mixture and to introduce the samples into the mass spectrometer. The peptide mixture was directly loaded on an analytical column (nanoACQUITY™ UPLC™ column, BEH300 C18, 1.7 mm, 75 mm_200 mm, Waters). Separation of peptides for IMS^E^ (MS^E^ with ion mobility separation) was done with a 90 min gradient from 5% buffer B to 40% buffer B. All MS^E^ analyses were performed as previously described^3^. The only modification was, that the collision energy was alternated between 4 eV in the precursor ion trace and a ramp 25–45 eV for fragment ion trace. Wave velocity was ramped from 1,000–800 m/s, wave height was set to 40 V.

LC-IMS^E^ data were processed using PLGS v2.5. Processing parameter were set as follows: Chromatographic peak width and MS TOF resolution were set to automatic, lock mass charge 2 set to 785.8426 Da/e with a lock mass window of 0.25 Da, low energy threshold 200.0 counts, elevated energy threshold 20.0 counts, intensity threshold 750 counts. The data were searched against a randomized Uniprot *S. aureus* COL database (version January 2011) with added laboratory contaminants and yeast ADH1 sequence (5,446 entries). For positive protein identification the following criteria had to be met: 1 fragment ion matched per peptide, 5 fragment ions matched per protein, 1 peptide matched per protein; 2 missed cleavages allowed, primary digest reagent: trypsin, fixed modification: carbamidomethylation C (+57.0215), variable modifications: deamidation N, Q (+0.9840), oxidation M (+15.9949), pyrrolidonecarboxylacid N-TERM (−27.9949). The protein false discovery rate (FDR) was set to 5%. For the final analysis only identifications based on at least two peptides were considered. A protein had to be identified in at least two out of three technical replicates per biological replicate. In addition, for the final analysis protein had to be present in two out of three biological replicates per time point which reduced FDR on protein level to less than 0.7%. The mass spectrometry proteomics data have been deposited to the ProteomeXchange Consortium ( http://proteomecentral.proteomexchange.org) via the PRIDE partner repository^52^ with the dataset identifier PXD000330.

Data generated in the LC-IMS^E^ mode were corrected for detector saturation effects by implementing a correction factor based on the ion accounting output files that were created for each sample by the PLGS software. The correction factor (cf) was calculated using the following equation.


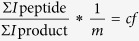


where Σ*I*peptide and Σ*I*product are the matched peptide/product intensity sums, and 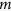 is the median of the ratios 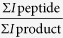 calculated for every protein quantified in a sample.

### Determination of protein copies per cell

For the calculation of absolute protein numbers per cell we determined the correlation between number of bacterial cells and optical density along the growth. Cell counting was performed in four technical replicates using light microscopy as described earlier^19^. Optical density was measured at a wavelength of 500 nm. The resulting correlation curve was used to calculate cell counts for each sample. Cell volume, numbers of cells in the process of cell division, and cell disruption efficiencies were determined to compensate variations between the different sampling time points (see above). Corrected protein counts have been used to calculate molecules per cell for each quantified protein.

### Preparation of samples for metabolomics

For intracellular metabolome analysis 20 mL cell suspension were sampled by using the protocol described previously^53^. The bacterial cells were filtered and washed using a vacuum filter system. Quenching took place immediately in liquid nitrogen, followed up by cell disruption, metabolite extraction and lyophilization of the intracellular metabolite extractions. For extracellular metabolome analysis 2 mL cell suspension were sterile filtered on ice by using a 0.45 μm pore size filter (Sarstedt AG, Nürnberg, Germany). Lyophilized intracellular und sterile filtered extracellular metabolome samples were stored at −20 °C until analysis.

### Analysis of the exometabolome

^1^H-NMR analysis and quantification of the extracellular metabolites was carried out as described recently^54^. Briefly, 400 μL of the sterile filtered culture supernatant was mixed with 200 μL of a 0.2 M sodium hydrogen phosphate buffer solution made up with 50% D_2_O which provides a nuclear magnetic resonance (NMR)-lock signal. NMR buffer solution also contained 1 mM TSP (3-trimethylsilyl-[2,2,3,3-D_4_]-1-propionic acid) as the internal standard. Absolute quantification of extracellular metabolites was done by integrating and referencing the proton signal areas of extracellular compounds to the TSP integral with its known numbers of protons and concentration.

### Analysis of the endometabolome

Intracellular metabolites were analyzed by both LC-MS and GC-MS, therefore at every sampling time point 20 mL cell suspension were sampled twice, once with 100 nmol Brom-ATP as internal standard for the LC-MS analysis and once with 20 nmol ribitol/norvaline as internal standards for the GC-MS analysis added into the extraction solution. LC-MS analysis was performed, as described earlier^55^, with lyophilized samples redissolved in 100 μL ultrapure water. For the GC-MS analysis lyophilized samples were derivatized with 60 μL MeOX (O-methylhydroxylamine-hydrochloride) for 90 min and with 120 μL MSTFA (N-methyl-N-(trimethylsilyl)trifluoroacetamide) for 30 min. Parameters of the GC-MS method were used as described previously^56^. Identification of metabolites based on m/z spectra and retention time of pure standard compounds. Analysis and interpretation of the LC-MS and GC-MS data of three biological replicates was carried out as described previously^30^.

## Additional Information

**How to cite this article**: Zühlke, D. *et al*. Costs of life - Dynamics of the protein inventory of *Staphylococcus aureus* during anaerobiosis. *Sci. Rep.* **6**, 28172; doi: 10.1038/srep28172 (2016).

## Supplementary Material

Supplementary Information

## Figures and Tables

**Table 1:** Intracellular nucleotide concentrations (nmol/mg CDW).

	ATP (AXPs)	GTP (GXPs)	UTP (UXPs)	CTP (CXPs)
phase I (t_0_)	14.4 (20.4)	2.0 (2.4)	5.8 (9.1)	2.8 (5.2)
phase II (t_1_–t_13_)	12.1 (15.4)	1.1 (1.2)	2.4 (3.9)	1.6 (2.6)
phase III (t_19_–t_24_)	1.3 (3.4)	0.5 (0.7)	1.0 (6.5)	0.7 (3.2)

For phase II and III mean values of concentrations from the different sampling time points were determined. Calculation of the sum of the adenosine (AXPs), guanosine (GXPs), uridine (UXPs) and cytidine (CXPs) nucleotides was done based on the mean concentrations of the respective mono-, di- and triphosphates.

**Table 2:** Stoichiometry of selected protein complexes from exponentially growing *S. aureus* cells were determined based on absolute protein quantification data obtained with the LC-IMS^E^ approach.

Protein complex	*S. aureus*	Reported stoichiometry	Reference^a^
SufB:SufC:SufD	1:2:1	1:2:1^b^	42
AtpA:AtpD:AtpG:AtpH:AtpC	6:5:1:1:1^c^	3:3:1:1:1^d^	57
RpoB:RpoA	1:2	1:2^b^	58
PdhA:PdhB:PdhC	1:1:1	1:1:1	59

^a^Reference for known stoichiometry.

^b^Shown for *E. coli.*

^c^The calculated molecule numbers lead to a different stoichiometry of the F1 complex as published because free subunits that are not integrated into the F1 complex may influence these values.

^d^Shown for bovine mitochondria.
