# Supplementary material for: Costs of life - Dynamics of the protein inventory of Staphylococcus aureus during anaerobiosis
Source: Sci Rep. 2016 Jun 27;6:28172. doi: 10.1038/srep28172 (PMC4921807; doi:10.1038/srep28172)
Supplement: Supplementary Information [file srep28172-s1.pdf]

Supplementary Information

Costs of life - Dynamics of the protein inventory of *Staphylococcus aureus* during anaerobiosis

Daniela Zühlke[1#], Kirsten Dörries[2#], Jörg Bernhardt[1], Sandra Maaß[1], Jan Muntel[1§], Volkmar Liebscher[3], Jan Pané-Farré[1], Katharina Riedel[1], Michael Lalk[2], Uwe Völker[4], Susanne Engelmann[1,5,6], Dörte Becher[1], Stephan Fuchs*[1,7], Michael Hecker[1]

[1]Institute of Microbiology, Ernst-Moritz-Arndt-University Greifswald, F.-L.-Jahn-Strasse 15, D-17487 Greifswald, Germany

[2]Institute of Biochemistry, Ernst-Moritz-Arndt-University Greifswald, Felix-Hausdorff-Strasse 4, D-17487 Greifswald, Germany

[3]Department of Mathematics and Informatics, Ernst-Moritz-Arndt-University Greifswald, Walther-Rathenau-Strasse 47, D-17487 Greifswald, Germany

[4]Interfaculty Institute for Genetics and Functional Genomics, Ernst-Moritz-Arndt-University Greifswald, F.-L.-Jahn-Strasse 15 a, D-17487 Greifswald, Germany

[5]Institute of Microbiology, Technical University Braunschweig, Inhoffenstrasse 7, D-38124 Braunschweig, Germany

[6]Helmholtz Institute for Infection Research, Microbial Proteomics, Inhoffenstrasse 7, D-38124 Braunschweig, Germany

[7]Robert Koch Institute, FG13 Nosocomial Pathogens and Antibiotic Resistance, Burgstrasse 37, D-38855 Wernigerode, Germany.

[§] Current address: Departments of Pathology, Boston Children's Hospital and Harvard Medical School, and the § Proteomics Center, Boston Children's Hospital, Boston, MA, USA

[#] Contributed equally

*Corresponding author:

Stephan Fuchs phone: +49 30 18754 4338
fax: +49 30 18754 4317
email: FuchsS@rki.de

CONTENTS

Supplementary Methods **3**

Fluorescence microscopic analysis of cell division and cell size determination. *3*

Pulse and pulse chase labeling experiments to detect differences in cell disruption efficiencies. *3*

Statistical analysis of proteome data. *4*

Statistical analysis of metabolome data. *5*

Supplementary Tables **6**

Supplementary Table S1. .... *6*

Supplementary Table S2. .... *9*

Supplementary Table S3. .... *9*

Supplementary Figures **10**

Supplementary Figure S1 .... *100*

Supplementary Figure S2 .... *11*

Supplementary Figure S3 .... *12*

Supplementary Figure S4 .... *13*

Supplementary Figure S5 .... *14*

Supplementary Figure S6 .... *15*

Supplementary Figure S7 .... *16*

Supplementary References **17**

Supplementary Methods

Fluorescence microscopic analysis of cell division and cell size determination. The cell membrane of *S. aureus* COL was stained using Nile Red (Life Technologies, Carlsbad, CA, USA). For staining with Nile Red, a sample of 1 mL cell culture was pelleted and subsequently resuspended in 1 mL synthetic medium supplemented with 0.7 µg mL^{-1} Nile Red. After incubating the cells for 5 minutes at room temperature they were pelleted and resuspended in 0.8 mL fresh synthetic medium. 5 µL of the samples were placed on a thin layer of 1.5% agarose in phosphate-buffered saline (PBS) which was mounted on an object slide. Images were acquired using a fluorescence microscope (Imager M2, Zeiss, Oberkochen, Germany) equipped with an EC-Plan-Neofluar objective (100x/1.3 oil ∞/0.17; Zeiss, Oberkochen, Germany) and the filter set 63 HE (ex BP 572/25, bs FT590, em BP629/62). Cell size determination and analysis of septum formation was done using ZEN v1.0.1.0 (Zeiss, Oberkochen, Germany). The volume of *S. aureus* cells was calculated using the equation for prolate spheroids; to determine the inner volume a cell wall thickness of 30 nm was assumed [1].

Fraction of cells with an already formed septum was measured by fluorescence microscopy using the membrane stain Nile Red. This was done for each sample by visual control of 450 cells (150 cells in 3 biological replicates). As expected, under exponential growth conditions about 25% of all cells are in the process of cell-division. Under anaerobic conditions the portion of dividing cells is lower (Supplementary Table S1). The percentage of dividing cells was used to adapt the cell numbers obtained from the correlation curve. Finally, the protein stability (pulse-chase labelling) was analyzed (see below).

No reliable difference in cell volume has been seen during aerobic and anaerobic growth (Supplementary Table S1).

Pulse and pulse chase labeling experiments to detect differences in cell disruption efficiencies. For radioactive pulse experiments *S. aureus* COL was grown in CDM medium and labelled with L-[^{35}S]-methionine (150 µCi/10 mL culture) for 5 min followed by the addition of 1 mL stop solution (10 mM

L-methionine, 3.1 mM chloramphenicol). Cells were labeled at OD_{500} = 0.5 (t0) and 1, 5, 7, 9, 13, 19, 24 h after shift to anaerobic conditions. For the pulse chase approach cells were grown until mid-log phase (OD_{500nm} 0.5) and labeled with L-[^{35}S]-methionine (1.5 mCi/100 mL culture) for 15 min followed by a chase with a 600,000 fold molar excess of cold methionine (10 mM final concentration). Samples were taken immediately after chase and 1, 5, 7, 9, 13, 19, 24 h after shift to anaerobic conditions. Samples were centrifuged (8,500 rpm 4°C, 5 min) and washed twice with TE buffer. Pellets were resuspended in 300 µL TE buffer. Five µL of the resuspended cells were directly spotted onto filter-paper discs to measure the incorporated radioactivity in whole cells. Additionally, the incorporated radioactivity in the cytoplasmic fraction was analyzed after cell disruption using Ribolyser (see below). Determination of incorporated radioactivity was carried out according to a standard procedure [2].

When we compared the incorporated radioactivity in whole cells and in cytosolic extracts we observed an unexpected increase (about 1.6-fold) in the incorporated activity in the cytosolic extract from time point t_0 to time point t_5 which can only be explained by increased cell disruption efficiency under anaerobic conditions. To exclude this effect the sample-specific ratio of incorporated radioactivity obtained from cytoplasmic extracts and from whole cells was used for cell count correction.

Statistical analysis of proteome data. Statistical analysis of proteome data is based on calculated absolute protein amounts (fmol/ng). LC-IMS^E was done with 3 technical and biological replicates for each time point, yielding a total of 9 measurements per time point. Only proteins which were observed in a minimum of two technical and biological measurements and present in all samples were included in the statistical analyses. Statistical tests were based on a protein-wise mixed effects model for the logarithmic protein amounts [3]. The deterministic effects give the mean level of the protein per time point. The random effects account for variability in level between biological replicates, for each time point separately. For each protein and data type an ANOVA test was

performed. Thus, a positive result indicates that there was a significant change in the mean levels over the time course (Supplementary Data 1). To account for multiple testing p-values were corrected using the false discovery rate approach of Benjamini and Hochberg [4]. Specifically, we estimated the q-values [5] with the method from Strimmer [6]. The analyses were implemented in the software R (version 2.15) with the additional packages “nlme” and “fdrtool”.

Statistical analysis of metabolome data. Experimental data are expressed as the mean of triplicate samples with its standard deviation. Visualization of time-course changes in concentrations of metabolites was done by using VANTED v2.01 and Microsoft Excel 2007 [7]. Statistically significant differences referred to the sample time point t_0 were calculated by using unpaired T-test with $*p \leq 0.05$ using VANTED v2.01 [7]. Time-courses of concentration of all identified extracellular and intracellular metabolites are displayed via a hierarchical clustered heatmap created by using MeV v4.8.1 with the following settings: optimized gene leaf order, Euclidean distance metric and average linkage method [8].

Supplementary Tables

Supplementary Table S1. Protein molecules per cell were calculated based on absolute protein quantification data obtained with the LC-IMS^E approach, cell numbers, cell volume, and proportion of dividing cells.

	t_0	t_5	t_7	t_9	t_{24}
protein molecules/cell[a]	1,170,000	990,000	820,000	1,020,000	2,540,000
SEM[b]	289,000	221,000	240,000	494,000	840,000
cell volume [µm³]	0.32	0.27	0.25	0.25	0.28
cells with septum formation [%]	25.56	20.67	15.56	14.00	7.33

[a] considering only proteins with predicted cytosolic localization; rounded to full thousand
[b] standard error of the mean of protein molecules per cell; rounded to full thousand

Supplementary Table S2. Abundance of transcriptional regulators in *S. aureus* COL during anaerobiosis.

protein	copies/cell				
	t_0	t_5	t_7	t_9	t_{24}
SarA	5,300	3,900	3,000	5,000	11,100
MgrA	2,600	2,500	1,800	2,900	6,700
CodY	1,900	2,500	1,900	2,500	6,400
SACOL0727	1,800	1,600	1,400	1,700	4,500
SarR	1,600	1,700	2,600	1,500	3,900
CcpA	1,300	1,100	800	1,100	2,300
ArgR	900	600	600	800	1,500
YycF	800	1,000	1,100	1,100	3,000
SACOL1541	500	1,100	900	1,000	2,800
PurR	500	600	500	700	1,800
VraR	400	500	400	500	1,300
SACOL1904	400	100	50	50	-
SrrA	300	1,000	900	1,300	3,900
PyrR	300	800	600	900	2,200
SarS	300	300	200	300	500
LexA	300	200	200	200	500
SirR	200	200	200	200	700
IcaR	200	200	100	200	400
SACOL1611	200	200	100	200	300
SACOL0890	200	100	100	200	400
SarZ	100	300	200	300	800
PhoP	100	200	200	200	400
ArlR	100	200	200	100	400
SACOL1919	100	200	100	100	200
SACOL2650	100	100	100	100	400
SACOL1733	100	100	100	100	400
TcaR	100	100	100	100	400
SACOL1296	100	100	100	100	200
SACOL0072	100	100	50	100	200
SACOL2531	100	50	50	50	200
SACOL1107	100	50	-	-	-
SaeR	50	200	200	200	1,600
SpxA	50	100	100	100	500
SACOL0120	50	100	50	-	-
SACOL0731	50	100	-	50	100
CtsR	50	50	100	50	100
SACOL0716	50	50	50	50	200
SACOL2317	50	50	50	50	100
SACOL1905	50	50	50	-	200
CobB	50	50	50	-	100
SACOL2349	50	50	-	-	50
SACOL0420	50	-	-	-	-

Supplementary Table S2 continued

**protein**	**copies/cell**				
	t_0	t_5	t_7	t_9	t_{24}
GlnR	50	-	-	-	-
GapR	-	300	200	300	1,000
CzrA	-	300	200	200	500
SACOL2555	-	100	50	-	300
LacR	-	50	50	50	100
SACOL0201	-	50	50	-	-
SACOL1550	-	50	-	50	-
SACOL1301	-	50	-	-	50
HssR	-	50	-	-	-
NreC	-	50	-	-	-
SarV	-	50	-	-	-
AgrA	-	-	50	-	-
LytR	-	-	-	300	-
ArgR	-	-	-	50	-
SACOL1997	-	-	-	-	100

Supplementary Table S3. Copies per cell of cytosolic proteins of unknown function with at least two-fold induction during phase II.

protein	copies/cell				
	t_0	t_5	t_7	t_9	t_{24}
SACOL2132	50	100	100	100	400
SACOL0286	50	50	50	100	100
SACOL1793	50	100	100	100	300
SACOL2255	50	100	50	100	100
SACOL0279	50	100	100	100	100
SACOL0830	50	100	100	200	400
SACOL0569	50	100	100	100	300
SACOL2533	50	100	100	100	300
SACOL0456	50	100	100	100	300
SACOL1204	50	100	300		
SACOL0730	50	50	100	100	200
SACOL2591	50	50	50	100	100
SACOL0220	100	700	400	500	1,700
SACOL1971	100	600		50	50
SACOL2436	100	400	300	400	1,300
SACOL0834	100	200	200	200	700
SACOL0668	100	200	200	200	500
SACOL1958	100	300	200	200	700
SACOL0821	100	200	200	200	800
SACOL0399	100	400	200	300	1,000
SACOL0785	100	300	300	300	1,000
SACOL0619	100	200	50	50	300
SACOL2710	100	100	100		300
SACOL2605	100	100	100	100	400
SACOL2297	100	100	200	200	400
SACOL1116	100	100	200	200	800
SACOL0496	100	100	600	200	300
SACOL2163	200	800	500	500	1,600
SACOL2518	200	500	200	200	900
SACOL1460	200	400	300	400	1,000
SACOL1620	200	500	400	400	1,200
SACOL2667	400	900	600	1,000	2,600
SACOL0872	400	700	500	900	2,400

Supplementary Figures

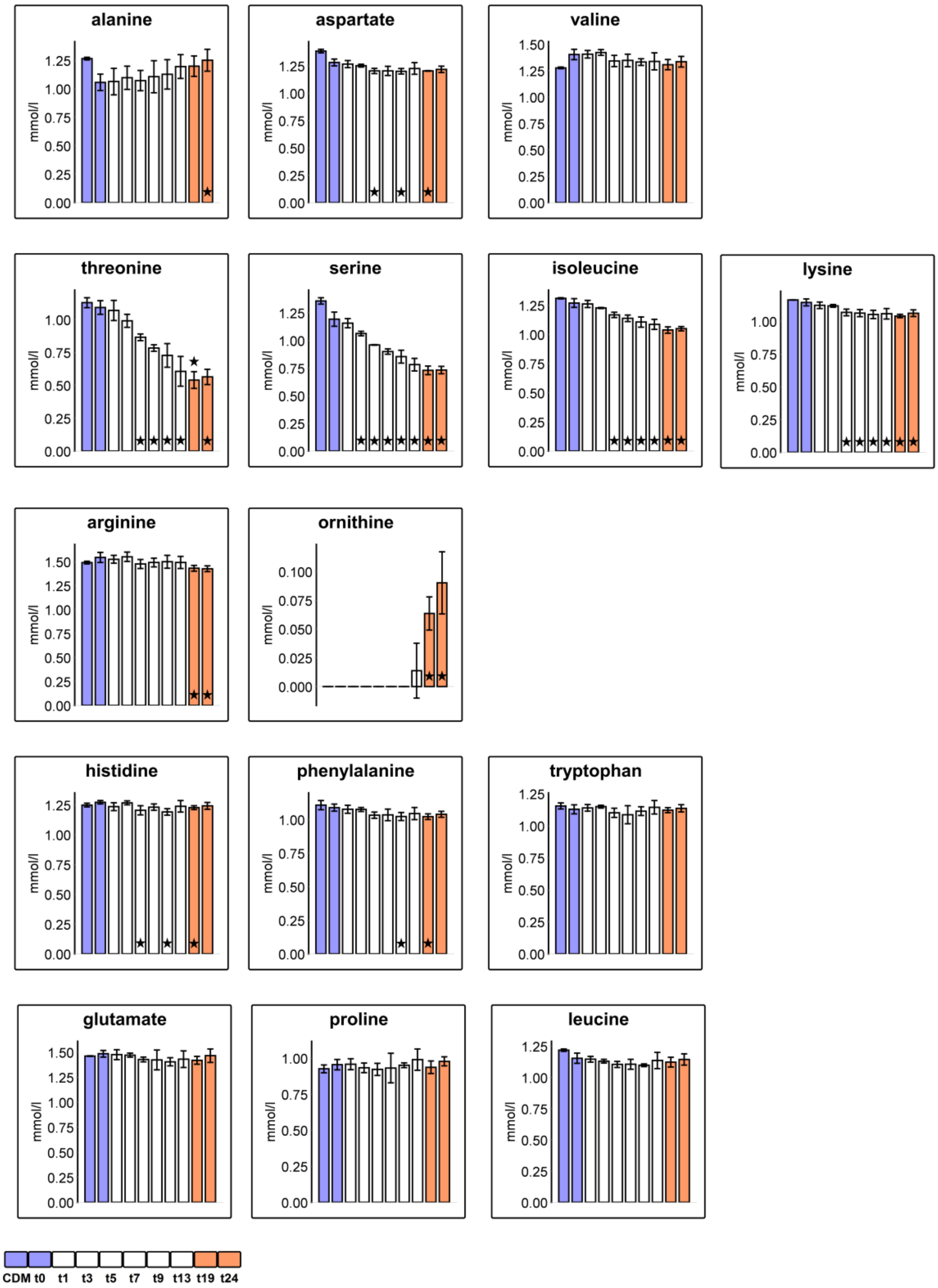

Supplementary Figure S1. Extracellular concentrations of amino acids during anaerobic growth of *S. aureus* [mmol/L]. The analysis of extracellular amino acids was conducted using ^{1}H-NMR spectroscopy. In aqueous solution cysteine reacts to a large part to cystine. For both metabolites no extracellular concentrations were evaluable due to strong pH dependent shifts in the NMR spectra. Bar colors indicate different physiological conditions: phase I (blue) aerobic in the presence of glucose; phase II (white) anaerobic in the presence of glucose; phase III (orange) anaerobic in the absence of glucose.

As a control the concentrations of the respective metabolites in the culture medium (CDM) is given (first blue bar in each diagram). Marked bars (*) indicate statistically significant changes with $p \leq 0.05$ compared to t_0 (unpaired t-test; see Supplementary Methods).

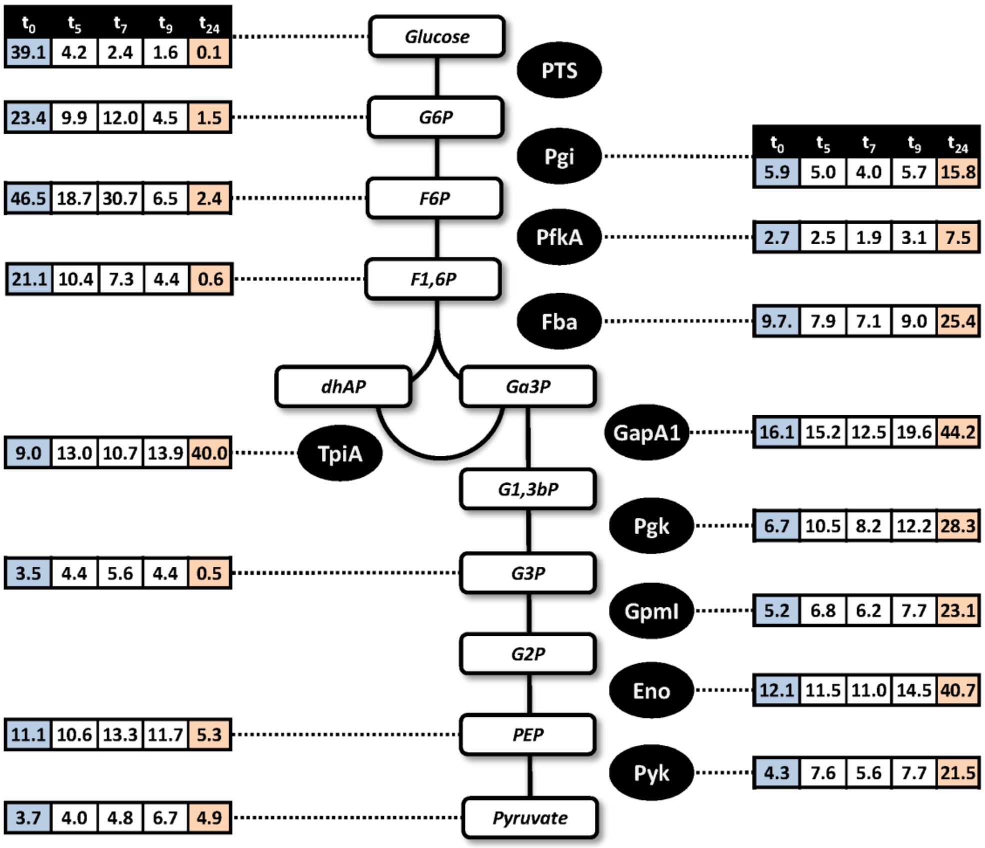

Supplementary Figure S2. Protein amounts and endometabolite concentrations related to the glycolysis of *S. aureus* COL upon anaerobic conditions. The absolute protein abundances (protein copies per cell; numbers are given in thousands) and the intracellular metabolite concentrations (in nmol/mg CDW) are shown for different time points (hours) after oxygen depletion. Colors indicate the different physiological conditions: phase I (blue) aerobic in the presence of glucose; phase II (white) anaerobic in the presence of glucose; phase III (orange) anaerobic in the absence of glucose. G6P = glucose 6-P, F6P = fructose 6-P, F1,6P = fructose 1,6-bP, dhAP = dihydroxyacetone-P; Ga3P = glyceraldehyde 3-P, G1,3bP = 1,3-bisphosphoglycerate, G3P = 3-phosphoglycerate, G2P = 2-phosphoglycerate, PEP = phosphoenolpyruvate, PTS = phosphotransferase system.

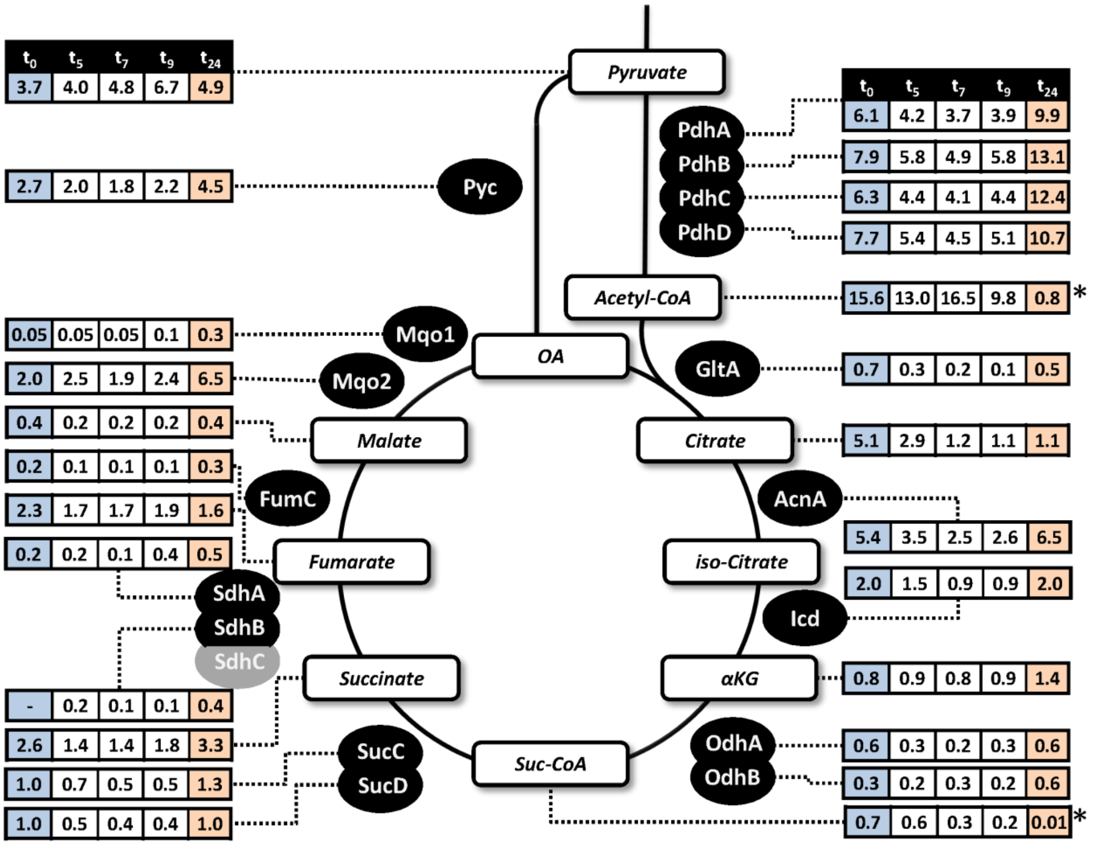

Supplementary Figure S3. Protein amounts and endometabolite concentrations related to the tricarbonic acid cycle in *S. aureus* COL upon anaerobic conditions. The absolute protein abundances (protein copies per cell; numbers are given in thousands) and the intracellular metabolite concentrations (in nmol/mg CDW) are shown for different time points (hours) after oxygen depletion. Proteins with grey background were not identified. Asterisks indicate metabolites that were provided with relative quantitative data [relative concentration/mg CDW]. Colors indicate different physiological conditions: phase I (blue) aerobic in the presence of glucose; phase II (white) anaerobic in the presence of glucose; phase III (orange) anaerobic in the absence of glucose. OA = oxalacetate, αKG = α-ketoglutarate, Suc-CoA = succinyl-CoA

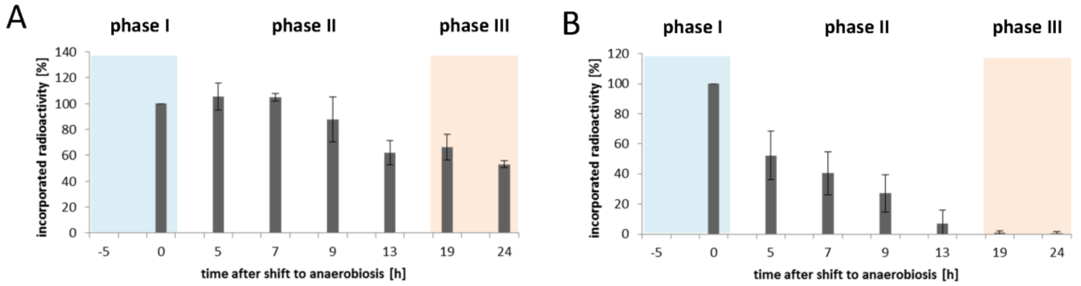

Supplementary Figure S4. Protein stability and protein synthesis in *S. aureus* COL under anaerobic conditions.

Protein synthesis rate (**A**) was determined by radioactive pulse labeling with L-[^{35}S]-Met at the indicated time points (see Supplementary Methods). Protein stability (**B**) was determined using pulse chase experiments with L-[^{35}S]-Met (see Supplementary Methods). The incorporated radioactivity was measured using whole cells. Bar colors indicate different physiological conditions: phase I (blue) aerobic in the presence of glucose; phase II (white) anaerobic in the presence of glucose; phase III (orange) anaerobic in the absence of glucose.

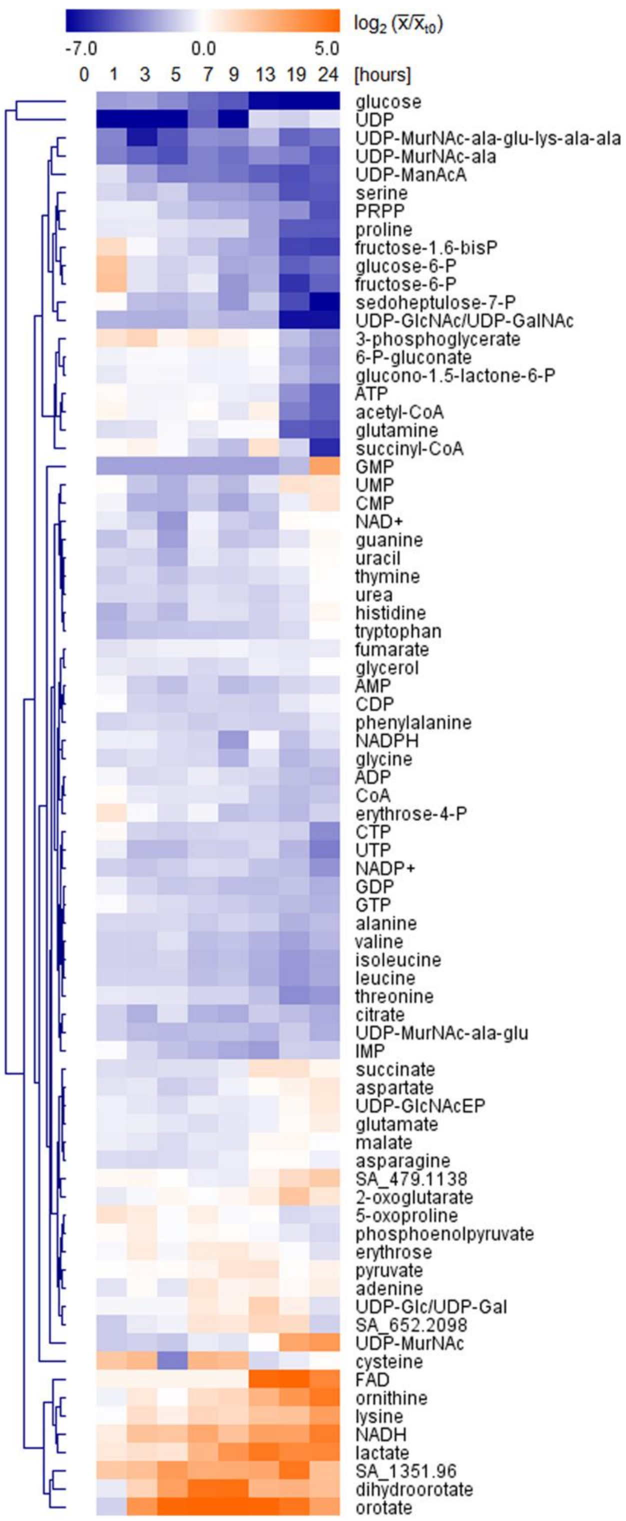

Supplementary Figure S5. Time-course changes of intracellular metabolites visualized by a hierarchically clustered heatmap. Displayed are the binary logarithm of the mean values of triplicate samples referred to t_0 as aerobic reference. Decreasing concentrations are colored in blue and increasing concentrations are colored in orange.

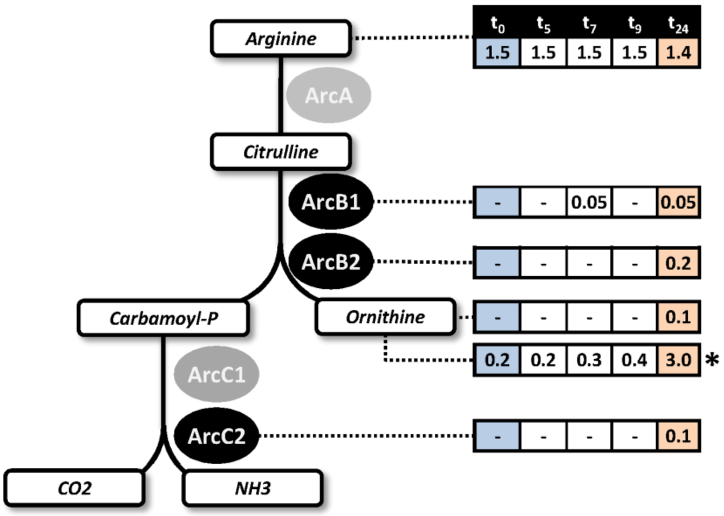

Supplementary Figure S6. Protein amounts and metabolite concentrations related to the arginine deiminase pathway in *S. aureus* COL upon anaerobic conditions. The absolute protein abundances (protein copies per cell; numbers are given in thousands), extracellular metabolite concentrations (in mmol/L), and intracellular ornithine concentrations (in nmol/mg CDW, marked with an asterisk) are shown for different time points (hours) after oxygen depletion. Proteins with grey background were not identified. Colors indicate different physiological conditions: phase I (blue) aerobic in the presence of glucose; phase II (white) anaerobic in the presence of glucose; phase III (orange) anaerobic in the absence of glucose.

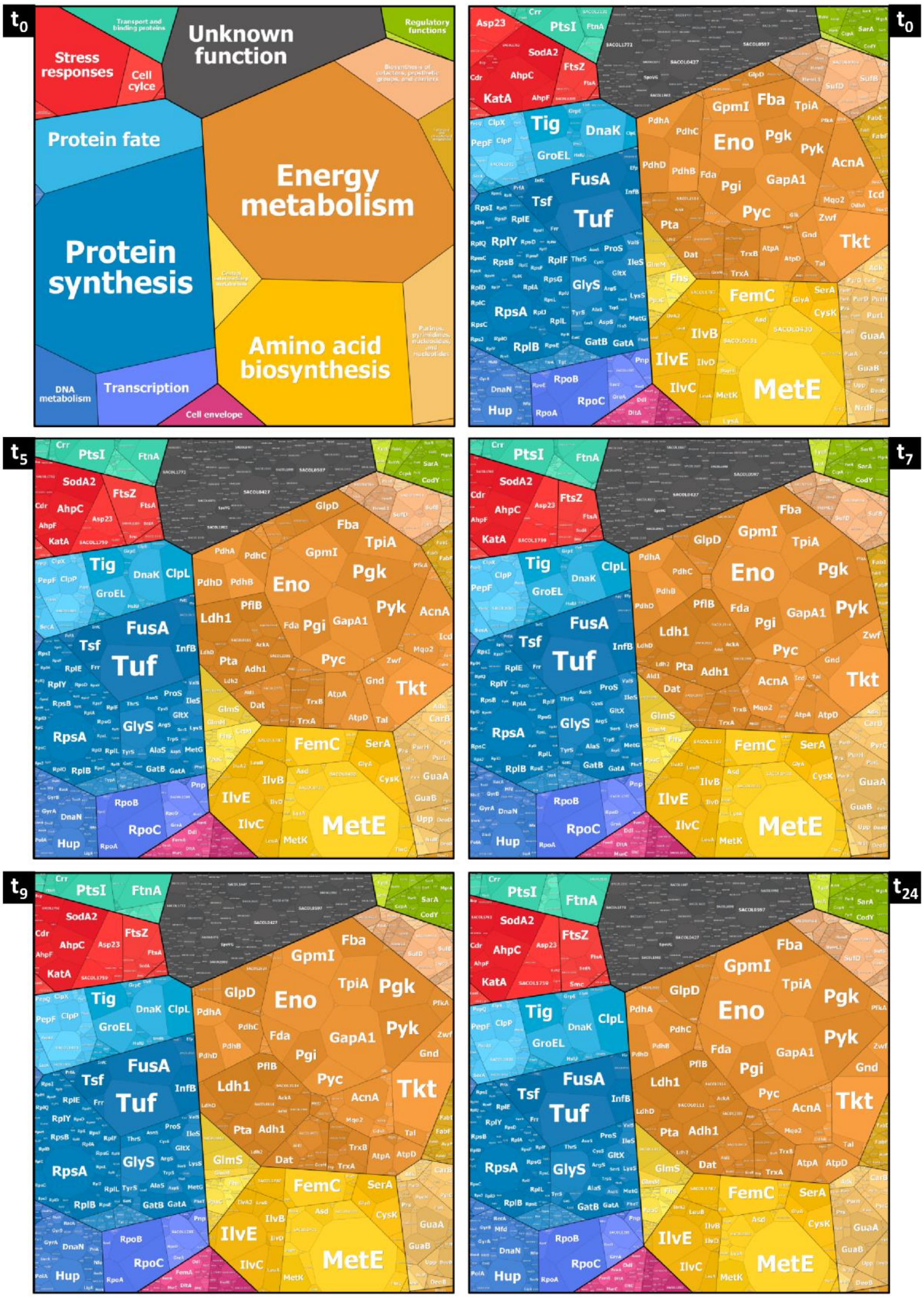

Supplementary Figure S7. Quantity-dependent visualization of investments in the protein inventory of *S. aureus* COL during long-term anaerobiosis. Investments were calculated by change in molecular count multiplied with molecular weight. Distribution of investments in specific cellular processes is shown in an area encoded fashion as Voronoi treemaps. The upper leftmost treemap shows functional protein classification based on TIGR Roles and manual curation. The different sample points can be grouped into phase I (aerobic growth in the presence of glucose; t_0), phase II (anaerobic growth in the presence of glucose; t_5, t_7, t_9), and phase IIII (anaerobi groth in the absence of glucose).

Supplementary References

1 Cui, L., Murakami, H., Kuwahara-Arai, K., Hanaki, H. & Hiramatsu, K. Contribution of a thickened cell wall and its glutamine nonamidated component to the vancomycin resistance expressed by Staphylococcus aureus Mu50. *Antimicrob Agents Chemother* **44**, 2276-2285 (2000).

2 Bernhardt, J., Buttner, K., Scharf, C. & Hecker, M. Dual channel imaging of two-dimensional electropherograms in Bacillus subtilis. *Electrophoresis* **20**, 2225-2240, doi:10.1002/(SICI)1522-2683(19990801)20:11<2225::AID-ELPS2225>3.0.CO;2-8 (1999).

3 Cui, X. & Churchill, G. A. Statistical tests for differential expression in cDNA microarray experiments. *Genome Biol* **4**, 210 (2003).

4 Benjamini, Y. & Hochberg, Y. Controlling the False Discovery Rate: A Practical and Powerful Approach to Multiple Testing. *Journal of the Royal Statistical Society. Series B (Methodological)* **57**, 289-300, doi:citeulike-article-id:1042553, doi: 10.2307/2346101 (1995).

5 Storey, J. A direct approach to false discovery rates. *Journal of the Royal Statistical Society: Series B (Statistical Methodology)* **64**, 479-498, doi:citeulike-article-id:1067590, doi: 10.1111/1467-9868.00346 (2002).

6 Strimmer, K. A unified approach to false discovery rate estimation. *BMC Bioinformatics* **9**, 303, doi:10.1186/1471-2105-9-303 (2008).

7 Klukas, C. & Schreiber, F. Integration of -omics data and networks for biomedical research with VANTED. *J Integr Bioinform* **7**, 112, doi:10.2390/biecoll-jib-2010-112 (2010).

8 Saeed, A. I. *et al.* TM4: a free, open-source system for microarray data management and analysis. *Biotechniques* **34**, 374-378 (2003).
